# Medicine and the future of health: reflecting on the past to forge ahead

**DOI:** 10.1186/s12916-016-0717-0

**Published:** 2016-10-25

**Authors:** Dale Fisher, Paul Wicks, Zaheer-Ud-Din Babar

**Affiliations:** 1 Infectious Disease Division, Department of Medicine, National University Hospital; National University Health Systems, 1E Kent Ridge Rd, Singapore, 119228 Singapore; 2 Yong Loo Lin School of Medicine, National University of Singapore, Singapore, Singapore; 3 PatientsLikeMe, Cambridge, MA USA; 4 School of Pharmacy, University of Auckland, Auckland, New Zealand; 5 Lahore Pharmacy College, Lahore, Pakistan

**Keywords:** Drugs, Pharmaceuticals, Future, Technology, Access, Patient-centred care, Antibiotics, Corruption, Drug shortages

## Abstract

The development of new therapies has a rich history, evolves quickly with societal trends, and will have an exciting future. The last century has seen an exponential increase in complex interactions between medical practitioners, pharmaceutical companies, governments and patients. We believe technology and societal expectations will open up the opportunity for more individuals to participate as information becomes more freely available and inequality less acceptable. Corporations must recognize that usual market forces do not function ideally in a setting where health is regarded as a human right, and as modern consumers, patients will increasingly take control of their own data, wellbeing, and even the means of production for developing their own treatments. Ethics and legislation will increasingly impact the processes that facilitate drug development, distribution and administration. This article collection is a cross-journal collaboration, between the *Journal of Pharmaceutical Policy and Practice* (*JoPPP*) and *BMC Medicine* that seeks to cover recent advances in drug development, medicines use, policy and access with high clinical and public health relevance in the future.

The Medicine and the Future of Health article collection is a joint collection between *BMC Medicine* and *Journal of Pharmaceutical Policy and Practice*. Therefore, this Editorial by the guest editors has been published in both journals.

Any vision of the future requires an appreciation of the past. Historically, the availability of medical treatments has paralleled life’s other “luxuries” and so was only available to the few. However, most remedies and would-be cures were not effective prior to the 20^th^ century, with few notable exceptions. Among these, digitalis, an extract from the purple foxglove (*Digitalis purpurea*) was first used in the dark ages as a poison until its discovery in 1775 for the treatment of heart failure [1]. More recently, the antimalarial drug artemether was extracted from the herb Qinghao, which had been used in China for over 2000 years [2].

In the 1920s, the emergence of more consistently effective pharmaceutical agents began, led by analgesics, including aspirin and morphine, insulin, and anti-infective agents such as sulphonamides and penicillin. However, it was soon realised that these potent new chemicals also carried risks. In 1937, investigators discovered, via a spate of reports to the American Medical Association, that an improperly prepared mixture of Elixir Sulfanilamide had killed over 100 people, prompting public outrage. This disaster led to the 1938 Federal Food, Drug, and Cosmetic Act to ensure that new drugs would be tested on animals and reviewed by the Food and Drug Administration. Subsequent amendments to the 1938 act introduced prescriptions for certain drugs (1951) and legislated for clinical trials (1962) [3]. Today, the post marketing surveillance of new medicines is much more sophisticated, and includes physician reports, patient outreach, Risk Evaluation and Mitigation Strategy programs, and the monitoring of electronic medical records. Such systems allow for enhanced safety via warning systems and the orderly withdrawal of drugs, though even this system suffers global inconsistencies [4].

As the pharmaceutical industry developed more effective medicines, quality of life of those suffering from many diseases clearly improved, as corticosteroids controlled inflammatory diseases, antihistamines controlled allergies, xanthines aided asthma patients, and options were offered to mental illness sufferers. Indeed, the human life span lengthened as infectious disease, heart disease, lung disease, and increasingly, cancer could be ameliorated through a combination of public health initiatives and better medicines. Today, medicines are intrinsic in all our lives, with almost half of US citizens taking a prescription medicine in the last 30 days [5]. However, rather than treating true disease pathology, the most intensively treated conditions at the population level in the US are pain, high cholesterol, depression and diabetes, arguably compensating for lifestyle changes brought about by dietary changes and a more sedentary lifestyle and due, in part, to the business models that encourage “blockbuster” drugs.

Medicines need to be readily available and affordable, however, unfortunately, there is an unacceptable level of medicine shortages across countries of all income levels. Currently, over 2 billion people do not have access to medicines [6]. In this context, the United Nations has set up a high-level forum to find ways to promote access to affordable medicines [7]. Iyengar et al. describe the driving factors behind access barriers and propose potential mitigation strategies [8].

There is widespread recognition that the existing global systems for innovation and access to medicines need reform. “Market failures” prevent new drugs from being developed that would primarily benefit the global poor, while factors such as high prices of medicines, weak health systems, corruption, and a lack of transparency, hinder efforts to distribute the medicines already available [9]. Community pharmacy triage services are emerging as a potential solution in a number of countries [10].

Access to medicines was once considered an issue confined to low- and middle-income countries; however, it is increasingly clear that access issues are also prevalent in high-income countries. For example, in the European Union, almost 50 % of drug expenditure is on cancer drugs [11, 12]. In the future, it is likely that access may be improved for cheaper generic options, but concerns remain over whether “biosimilars” will successfully fill the same role [13].

Antibiotics, as a class, and their usage warrant consideration in their own right, especially given that their efficacy relies partly upon the extent of their use in other patients. Dyer et al. showed that one-third of US antibiotic prescriptions are inappropriate, and he warns us that we may be taking our once potent antibiotics for granted [14]. Because the drug targets and the class itself originate from nature, Woon et al. make the case that we must consider the effective use of antibiotics as akin to managing a delicate ecosystem [15].

Ironically, given their overuse in the US, antibiotics are a case study in the difficulty of access to drugs in low- and middle-income countries. Articles to be included in *JoPPP* will consider the national and global strategies needed to improve access, raise antibiotic quality, diffuse accurate diagnostics to the point of care, and ensure local stewardship for the sustainable optimisation of antibiotic use [16]. The understanding of these factors and usefulness of interventions through incentives and legislation will continue to evolve. Further affecting the usage of antibiotics will be more rapid point-of-care diagnostics, vaccines, faecal microbiota transplantation, probiotics and other novel approaches to infectious diseases. It is unlikely, however, that alternative technologies will displace the need for the conventional class and its development.

Looking forward, IMS Health has predicted that global spending on pharmaceuticals will increase by 30 % from 2015 to 2020, to US$ 1.4 trillion, due in part to improved access, breakthrough innovations and cheaper drugs. A large portion of the growth is also occurring in India, China, Brazil and Indonesia, the so called “pharmemerging markets”, in contrast to the US, where they predict more than 90 % of drugs purchased will be generics [17].

“Unfairness” in health is becoming increasingly unacceptable as a social norm. People should not be treated differently because of individual demographic factors; with the right information in hand, patients will increasingly be treated according to their need as Norheim et al. explore [18]. Such “unfairness” within countries is easier to address compared to global health corruption, a covert exploitation of people and resources which will require concentrated efforts to expose. In a forum article, Mackey and a multidisciplinary panel discuss the ways in which corruption affects global health at all levels, and explore the potential solutions in a post-2015 development agenda [19].

In the future, it is very likely that the use of medicines will be greatly influenced by technology, consumer education and self-awareness regarding lifestyle and diseases [20]. Technology will be a key driver for change in the future, enhancing the medical skillset of healthcare professionals facilitating updates and change in parallel with consumers [21]. This could influence the way people use medicines and how healthcare professionals manage patients. Novel change could include tailor-made drugs on the basis of pharmacogenomic data or medicines manufactured locally and on demand through 3D printing [22]. Future personalised sensors could measure clinical parameters and blood biomarkers transmitting data in real time to a cloud or, for instance, sending alerts when a stroke is in its earliest stages [23].

We will continue to feature articles addressing how medicine might be expected to evolve to be more individual patient centred. A forthcoming Forum article from leading thinkers in the area will consider the opportunity for the “data shadow” of smartphones to aid in detecting depression, for patient-reported outcomes to improve self-management, for clinical trials to make their quantum leap, and for the value perceived by patients to flow back into the learning health system, perhaps supported by new forms of machine learning.

Beyond the colourful history that belongs to the emergence of pharmaceuticals in the last century there will be an ongoing evolution in response to changing needs driven largely by consumer demand and expectations. Implicit in the very commission of this body of work risks supporting a paternalistic notion that “experts” can set the agenda for the future of medicine. The truth is quite the opposite where, in this era of information, “citizen health hackers” [24] may lead patients to self-experiment at a faster pace than traditional players via digital platforms and a mantra to experiment beyond the traditional confines of medicine. Control of the music industry and the lay press has moved to the consumer. Such a move in the field of medicines might lead to accelerated discoveries and innovation that could theoretically outpace the entrenched players providing consumer benefits via lower prices and more rapid access as well as global equity. If we let patients help, they may well lead us into the future of medicine.
